# The effect of the development of an emergency transfer system on the travel time to tertiary care centres in Japan

**DOI:** 10.1186/1476-072X-5-25

**Published:** 2006-06-04

**Authors:** Makiko Miwa, Hiroyuki Kawaguchi, Hideaki Arima, Kazuo Kawahara

**Affiliations:** 1 Department of Health Policy Science, Graduate School of Tokyo Medical and Dental University, Tokyo, Japan; 2 Department of Health Services Management, Graduate School of International University of Health and Welfare, Tokyo, Japan

## Abstract

**Background:**

In Japan, the emergency medical system is categorized into three levels: primary, secondary, and tertiary, depending on the severity of the condition of the patient. Tertiary care centres accept patients who require 24-h monitoring. In this research, the average travel times (minutes) from the centroids of all municipalities in Japan to the nearest tertiary care centre were estimated, using the geographic information system. The systems affecting travel time to tertiary care centres were also examined. Regression analysis was performed to determine the factors affecting the travel time to tertiary care centres, using selected variables representing road conditions and the emergency transfer system. Linear regression analysis was performed to identify specific benchmarks that would be effective in reducing the average travel time to tertiary care centres in prefectures with travel times longer than the average 57 min.

**Results:**

The mean travel time was 57 min, the range was 83 min, and the standard deviation was 20.4. As a result of multiple regression analysis, average coverage area per tertiary care centre, kilometres of highway road per square kilometre, and population were selected as variables with impact on the average travel time. Based on results from linear regression analysis, benchmarks for the emergency transfer system that would effectively reduce travel time to the mean value of 57 min were identified: 26% pavement ratio of roads (percentage of paved road to general roads), and three tertiary care centres and 108 ambulances.

**Conclusion:**

Regional gaps in the travel time to tertiary care centres were identified in Japan. The systems we should focus on to reducing travel time were identified. Further reduction of travel time to tertiary care centres can be effectively achieved by improving these specific systems. Linear regression analysis showed that a 26% pavement ratio and three tertiary care centres are beneficial to prefectures with an average time longer than the mean score, to achieve a reduction of travel time. Measures for reducing travel time need to be considered in policy-making to re-evaluate the current locations of tertiary care centres to provide equality of access to emergency medicine.

## Background

In Japan, the emergency medical system has been provided systematically as a result of the Medical Care Law enacted in 1985, to ensure that "anyone can receive appropriate emergency medical care anytime, anywhere". The actual framework and infrastructure of emergency medical care have been developed through Medical Care Planning, which is ruled by Medical Care Law to establish the provision of the health care system in Japan. Medical Care Planning specifically states the requirement of, "securing and maintaining the emergency medical care system" [1]. In accordance with the framework provided by Medical Care Planning, the emergency medical system in Japan is categorized into three levels: primary, secondary, and tertiary, depending on the severity of the trauma and/or condition of the patients. Tertiary care centres accept patients whose conditions are life-threatening, or require 24-h monitoring. As of 1 February 2005, there are 175 tertiary care centres in Japan [2]. "Tertiary care centres" must receive the prefecture governor's approval in order to operate as such. The Ministry of Health, Labour and Welfare (MHLW) has established the standard that at least one tertiary care centre should be located for every million of the population [3]. However, there are regional gaps in the number of tertiary care centres per million capita [4]. Over-populated metropolitan areas such as Tokyo and Osaka satisfy the established standard (1.75 and 1.15 centres per million capita, respectively), whereas under-populated areas such as Akita prefecture, located in the north of mainland Japan, and Kagoshima prefecture, located in the south, fall short of the standard (0.85 and 0.56 centres per million capita, respectively) [4]. This is a result of the historical process in which tertiary care centres were developed, which focused on pure quantity, or numbers of centres, as a benchmark. Therefore, it is estimated that there is a regional gap in the accessibility to tertiary care centres in Japan.

As tertiary medical care targets patients with serious conditions, travel time to the tertiary care centre has a significant impact on the survival rate of patients, especially when the condition involves cerebral haemorrhage, subarachnoid haemorrhage, acute myocardial infarction, acute heart failure, pneumonia, or cardio pulmonary arrest (CPA) [5]. However, there is very little data available on the actual travel time to tertiary care centres. Hashimoto et al. [5] identified that in Nagasaki prefecture, located in the south of Japan, the average travel time measured from the initial response to the emergency call to the arrival at the scene was 7.3 min, and the average travel time measured from the initial response to the emergency call to the arrival at the emergency hospital was 26.9 min. On the other hand, the Fire and Disaster Management Agency (FDMA), which oversees the emergency transfer system, publicized in 2003 that the average travel time measured from the initial response to the emergency call to the arrival at the scene was 6.3 min and from the initial response to the emergency call to the arrival at the emergency hospital via ambulance, was 29.4 min [6]. However, there is no detailed information on analysis by emergency level, or additional data on situations where the patient was transported by other means. Thus, the information provided by the FDMA is insufficient in terms of assessing accessibility to tertiary care centres.

It is estimated that the travel time to tertiary care centres is affected by the current road conditions [7,8] as well as the development of the emergency transfer system. Therefore, in this paper, we estimated the average travel time (minutes) to tertiary care centres in all prefectures in Japan and identified the regional gaps in the travel time to tertiary care centres using a geographic information system (GIS). We performed regression analysis to determine the factors associated with the travel time to tertiary care centres, using selected variables representing road conditions and the emergency transfer system. We have identified benchmarks for the emergency transfer system that would be effective in reducing the average travel time to tertiary care centres in prefectures with travel times longer than the average 57 min.

Historically, research on travel time in medical accessibility has utilized GIS, and a number of studies have been published on various types of health service. In studies on accessibility to general practitioners, Lovett et al. [9] examined the use of patient registers and GIS. Bamford et al. [10] examined accessibility to general practitioners using Accessibility/Remoteness Index for Australia (ARIA) as a tool for health service planning in Australia. With regard to studies of accessibility to tertiary hospitals, Christie and Fone [11] described hypothetical scenarios on changes in service provision in Wales. With regard to studies of health care planning, hospital service areas were examined using patient travel patterns in Switzerland [12]. However, in contrast to these papers which examine active situations using GIS, there are very few papers published in Japan that introduce GIS into the medical field; one study reported on the spatial distribution of the needs of healthcare using GIS [13]. In the nursing field, the location of visiting nurses and visiting care facilities were examined [14]. To our knowledge, no published literature has examined the relationship between the emergency transfer system and the travel time to tertiary care centres in all prefectures in Japan. Therefore, we believe our research proposes an innovative approach.

## Results

### Average travel time

**Figure 1:**
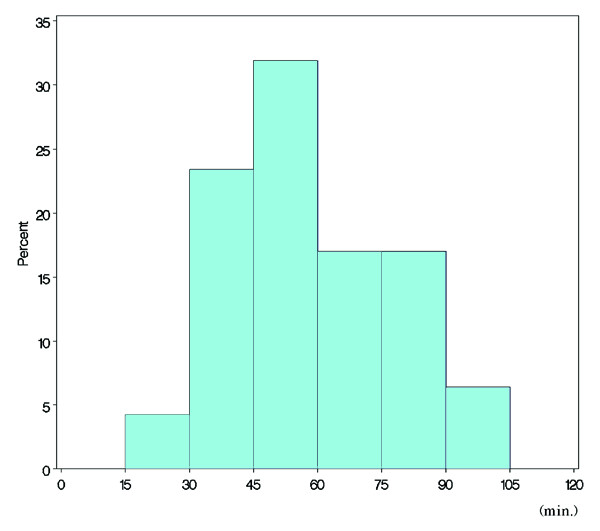
Histogram of average travel time (minutes).

**Figure 2:**
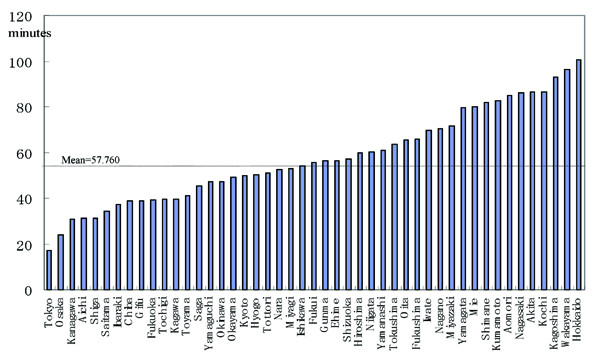
Average travel times (minutes) in all prefectures in Japan (*N *= 47).

**Figure 3:**
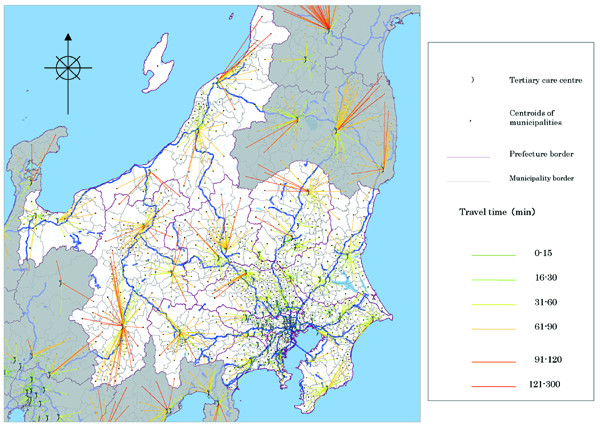
**Travel time (minutes) to the nearest tertiary care centre from centroids of municipalities**. This shows centroids in the Kanto area, which includes Ibaraki, Tochigi, Gunma, Saitama, Chiba, Tokyo, Kanagawa, Niigata, Toyama, Yamanashi and Nagano.

A histogram of average travel time (minutes) is shown in Figure 1. Table 1 shows the distribution of the average travel time. The distribution was nearly symmetrical. Tokyo had the shortest average travel time, at approximately 17 min; Hokkaido had the longest average travel time, at about 100 min (Table 1). Figure 2 shows the average travel times in all prefectures in Japan (*N *= 47), and Figure 3 shows the travel time in minutes to the nearest tertiary care centres from each of the centroids of municipalities in the Kanto area, using the map from the GIS software (scale 1 to 25,000).

**Table 1:** Distribution of average travel time and extreme values

Prefecture	Minimum (min)	Prefecture	Maximum (min)
Tokyo	17.0189	Akita	86.5952
Osaka	24.1818	Kochi	86.6596
Kanagawa	31.0000	Kagoshima	93.1228
Aichi	31.1319	Wakayama	96.2979
Shiga	31.2424	Hokkaido	100.5094
			
Mean (min)	57.76065	SD	20.42122
Median (min)	55.51724	Variance	417.0262
Mode		Range	83.49057
		Interquartile range	32.09164

SD, standard deviation.

### Population

**Figure 4:**
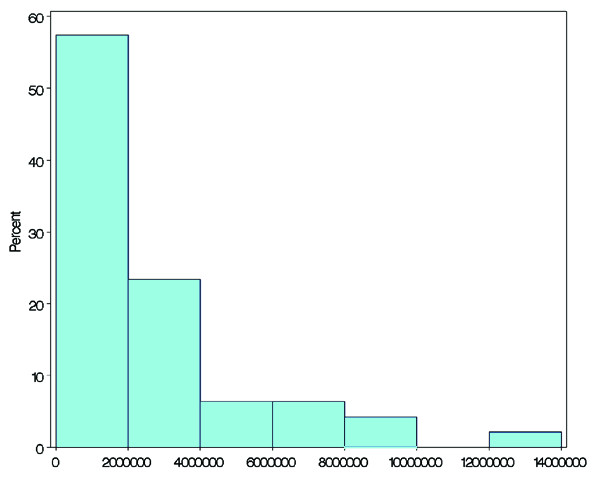
Histogram of population.

Population values are shown in a histogram (Figure 4) and the distribution, shown in Table 2, was nearly exponential. The least populated prefecture was Tottori, with about 600,000 people, and the most populous prefecture was Tokyo, with nearly 12,000,000 people (Table 2). The top four prefectures in terms of population, in descending order from Tokyo to Aichi, corresponded exactly to the prefectures with the smallest average travel time. Of the five prefectures with the smallest population, Kochi was the only prefecture ranked in the top five prefectures with the longest average travel time.

**Table 2:** Distribution of population (people) and extreme values

Prefecture	Minimum	Prefecture	Maximum
Tottori	609000	Saitama	7047000
Shimane	749000	Aichi	7192000
Kochi	803000	Kanagawa	8732000
Tokushima	813000	Osaka	8814000
Fukui	825000	Tokyo	12378000
			
Mean	2716745	SD	2571554
Median	1769000	Variance	6.61289-E12
Mode		Range	11769000
		Interquartile range	1716000

### Size (square kilometres)

**Figure 5:**
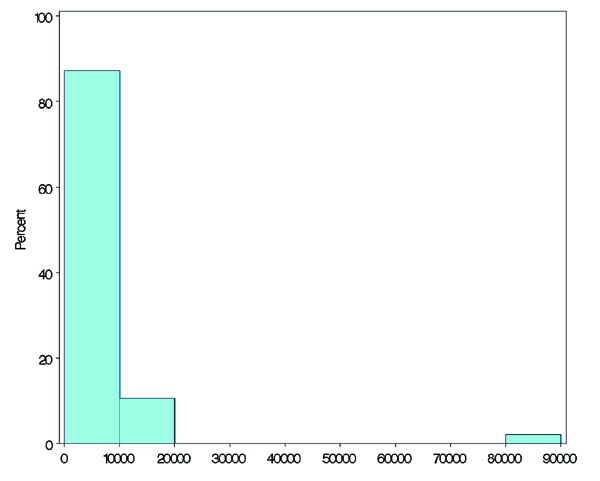
Histogram of size.

The size (km^2^) of each prefecture was also analysed (Figure 5, Table 3). The distribution was nearly exponential. The smallest prefecture was Kagawa, at about 1850 km^2^. The largest prefecture was Hokkaido, at nearly 83,500 km^2^; the size of Hokkaido is exceptional compared to other prefectures. As a result, Hokkaido had the longest average travel time (Table 3).

**Table 3:** Distribution of size (km^2^) and extreme values

Prefecture	Minimum (km^2^)	Prefecture	Maximum (km^2^)
Kagawa	1862	Akita	11434
Osaka	1894	Nagano	12374
Tokyo	2102	Fukushima	13783
Toyama	2132	Iwate	15279
Okinawa	2274	Hokkaido	83455
			
Mean	7739.149	SD	11701
Median	5761.000	Variance	136918475
Mode		Range	81593
		Interquartile range	3299

### Correlation between population and size (km^2^) with each variable

Pearson and Spearman correlation coefficients were performed (Table 4). The variables used for regression analysis of average travel time were selected based on the correlation analysis. We limited our regression analysis to variables with Pearson's or Spearman's correlation of less than 0.8, and those with meaningful interpretations (Table 4). This was done in order to avoid the situation where the effects of major information included in the variables would be counteracted by calculating the ratio, and result in emphasizing errors and producing false analysis.

**Table 4:** Correlation between population and size (km^2^) with each variable

	Pearson's correlation		Spearman's correlation	
	
	Population	Size (km^2^)	Population	Size (km^2^)
Population		-0.24		0.11
Average travel time (min)	-0.61	0.47	-0.50	0.54
Pavement ratio (%)	0.52	-0.55	0.03	-0.57
Size (km^2^)	-0.24		0.11	
Kilometres of general road	0.33	0.53	0.65	0.62
Kilometres of express road	0.05	0.68	0.44	0.64
Mountain area (km^2^)	-0.36	0.90^a^	-0.16	0.85^a^
Total kilometres of all roads	0.44	0.48	0.76	0.56
Road area (km^2^)	0.56	0.43	0.81^a^	0.49
Kilometres of impassable road	0.28	0.23	0.64	0.30
Maintenance ratio of general road (%)	0.19	-0.15	0.01	-0.18
Number of tertiary care centres	0.91^a^	-0.20	0.83^a^	0.02
Number of fire stations	0.95^a^	-0.06	0.88^a^	0.20
Percentage of emergency team with EMT to all emergency teams (%)	0.54	-0.31	0.37	-0.29
Number of ambulances	0.94^a^	-0.03	0.94^a^	0.23
Number of emergency teams	0.91^a^	0.02	0.93^a^	0.24
Number of emergency teams with EMT	0.97^a^	-0.14	0.90^a^	0.14
Number of EMT	0.97^a^	-0.21	0.92^a^	0.10
Number of emergency crews	0.76	0.15	0.89^a^	0.24
Number of emergency medical specialists	0.90^a^	-0.24	0.77	-0.03
Number of tertiary hospitals	0.89^a^	-0.22	0.75	-0.04
Number of secondary hospitals	0.94^a^	-0.20	0.88^a^	0.05
Number of primary hospitals	0.44	-0.03	0.41	-0.05

^a^Coefficient correlation of >0.8.

### Single regression analysis

Except for average travel time, none of the variables were adjusted for population or size (km^2^). The variable with Pearson's or Spearman's correlation of less than 0.8 and with meaningful interpretation, i.e., the "number of primary hospitals per million capita" was used to adjust for population. To adjust for size the same criteria as in the case of adjusting for population were used; the following variables were adjusted: "population density (population/size)", "kilometres of general road per square kilometre", "kilometres of highway road per square kilometre", "total kilometres of all roads per square kilometre", "kilometres of impassable road per square kilometre", "average coverage area per tertiary care centre", "average coverage area per fire station", "average coverage area per ambulance", "average coverage area per emergency team", "average coverage area per emergency team with EMT", "average coverage area per EMT", "average coverage area per emergency crew", "average coverage area per emergency medical specialist", "average coverage area per tertiary hospitals", "average coverage area per secondary hospitals", and "average coverage area per primary hospitals" (Table 5). For variables with significant difference before and after adjustment, the variable with the larger *t *value was adopted.

**Table 5:** Single regression analysis with average travel time and selected variables

Label	Parameter	Standard deviation	*t *value	Pr>|*t*|
Intercept	69.15509	3.36611	20.54	<0.0001
Population	-0.00000465	9.17E-07	-5.06	**<0.0001
				
Intercept	72.32474	6.86429	10.54	<0.0001
Pavement ratio (%)	-0.5484	0.22274	-2.46	0.0178*
				
Intercept	38.70193	5.68413	6.81	<0.0001
Size (km^2^)	0.00298	0.00083171	3.58	0.0009**
				
Intercept	60.64811	6.51694	9.31	<0.0001
Kilometres of general road	-0.00016857	0.00025751	-0.65	0.5161
				
Intercept	58.56759	5.69418	10.29	<0.0001
Kilometres of express road	-0.01191	0.03352	-0.36	0.7241
				
Intercept	41.20992	4.54893	9.06	<0.0001
Mountain area (km^2^)	0.00379	0.00092453	4.1	0.0002**
				
Intercept	63.69628	6.44123	9.89	<0.0001
Total kilometres of all roads	-0.00028853	0.00024217	-1.19	0.2399
				
Intercept	67.92296	6.74665	10.07	<0.0001
Road area (km^2^)	-0.0799	0.04414	-1.81	0.0771
				
Intercept	60.30891	3.97391	15.18	<0.0001
Kilometres of impassable road	-0.00097826	0.00077232	-1.27	0.2119
				
Intercept	88.52593	17.26037	5.13	<0.0001
Maintenance ratio of general road(%)	-0.59098	0.31753	-1.86	0.0694
				
Intercept	69.46632	3.25579	21.34	<0.0001
Number of tertiary care centres	-3.4803	0.64365	-5.41	<0.0001**
				
Intercept	72.04288	4.24031	16.99	<0.0001
Number of fire stations	-0.17113	0.03901	-4.39	<0.0001**
				
Intercept	103.57903	10.67744	9.7	<0.0001
Percentage of emergency team with EMT to all emergency teams (%)	-0.6629	0.14747	-4.5	<0.0001**
				
Intercept	73.85993	5.09866	14.49	<0.0001
Number of ambulances	-0.1485	0.0386	-3.85	0.0004**
				
Intercept	74.32109	5.35074	13.89	<0.0001
Number of emergency teams	-0.18252	0.04908	-3.72	0.0006**
				
Intercept	72.96964	3.992	18.28	<0.0001
Number of emergency teams with EMT	-0.22835	0.04579	-4.99	<0.0001**
				
Intercept	70.48444	3.81825	18.46	<0.0001
Number of EMT	-0.04839	0.01051	-4.6	<0.0001**
				
Intercept	74.14823	5.8457	12.68	<0.0001
Number of emergency crew	-0.01486	0.00449	-3.31	0.0019**
				
Intercept	64.71215	3.0713	21.07	<0.0001
Number of emergency medical specialists	-0.19617	0.04584	-4.28	<0.0001**
				
Intercept	70.70814	3.59755	19.65	<0.0001
Number of tertiary hospitals	-3.29038	0.65072	-5.06	<0.0001**
				
Intercept	68.89425	3.80355	18.11	<0.0001
Number of secondary hospitals	-0.1634	0.03912	-4.18	0.0001**
				
Intercept	61.56359	5.30389	11.61	<0.0001
Number of primary hospitals	-0.14026	0.13185	-1.06	0.2932
				
Intercept	48.80577	4.61801	10.57	<0.0001
Number of primary hospitals per million capita	4.4139	2.02837	2.18	0.035*
				
Intercept	63.54299	2.75577	23.06	<0.0001
Population density	-0.0099	0.00207	-4.79	<0.0001**
				
Intercept	79.81974	4.73202	16.87	<0.0001
Kilometres of general road per square kilometre	-5.4808	0.99273	-5.52	<0.0001**
				
Intercept	79.86274	5.00264	15.96	<0.0001
Kilometres of highway road per square kilometre	-890.52604	171.69149	-5.19	<0.0001**
				
Intercept	79.03227	4.29357	18.41	<0.0001
Total kilometres of all roads per square kilometre	-4.95391	0.82665	-5.99	<0.0001**
				
Intercept	64.5504	3.63863	17.74	<0.0001
Kilometres of impassable road per square kilometre	-11.8776	3.83601	-3.1	0.0034**
				
Intercept	38.47616	3.09223	12.44	<0.0001
Average coverage area per tertiary care centre	0.00662	0.0008726	7.59	<0.0001**
				
Intercept	36.56935	4.21284	8.68	<0.0001
Average coverage area per fire station	0.21018	0.03711	5.66	<0.0001**
				
Intercept	33.75986	4.73479	7.13	<0.0001
Average coverage area per ambulance	0.35238	0.06366	5.53	<0.0001**
				
Intercept	33.43733	4.71752	7.09	<0.0001
Average coverage are per emergency team	0.30365	0.05396	5.63	<0.0001**
				
Intercept	34.1383	3.9444	8.65	<0.0001
Average coverage area per emergency team with EMT	0.18787	0.02789	6.74	<0.0001**
				
Intercept	35.06809	4.1664	8.42	<0.0001
Average coverage area per EMT	0.70799	0.11604	6.1	<0.0001**
				
Intercept	30.7381	4.59037	6.7	<0.0001
Average coverage area per emergency crew	4.2571	0.6655	6.4	<0.0001**
				
Intercept	42.36434	3.63255	11.66	<0.0001
Average coverage area per emergency medical specialist	0.0437	0.00847	5.16	<0.0001**
				
Intercept	37.4911	3.71768	10.08	<0.0001
Average coverage area per tertiary hospitals	0.00917	0.00145	6.33	<0.0001**
				
Intercept	41.81954	4.094	10.21	<0.0001
Average coverage area per secondary hospitals	0.11199	0.02467	4.54	<0.0001**
				
Intercept	49.73788	3.74773	13.27	<0.0001
Average cover area per primary hospitals	0.02387	0.00873	2.73	0.009**

d.f. = 1 for all variables. *p < 0.05, **p < 0.01.

### Correlation between selected variables

We analysed the correlation between variables selected as in the previous section, and refined candidate variables for multiple regression analysis. If there was strong correlation between variables, it could give rise to the issue of multicollinearity, which would falsely impact the results of the analysis. Variables intercorrelated among themselves were shown in Pearson's correlation (Table 6) and Spearman's correlation (Table 7);

**Table 6:** Pearson's correlation among variables

**Pearson 's correlation**																						
Label	*Variables *(*Var*.)	**Var. 1**	**Var. 2**	**Var. 3**	**Var. 4**	**Var. 5**	**Var. 6**	**Var. 7**	**Var. 8**	**Var. 9**	**Var. 10**	**Var. 11**	**Var. 12**	**Var. 13**	**Var. 14**	**Var. 15**	**Var. 16**	**Var. 17**	**Var. 18**	**Var. 19**	**Var. 20**	**Var.21**
**Var. 1**	Population	1.00																				
**Var. 2**	**Pavement ratio (%)**	0.52	1.00																			
**Var. 3**	**Size(km^2^)**	-0.24	-0.55	1.00																		
**Var. 4**	**Mountain area(km^2^)**	-0.36	-0.48	a0.90	1.00																	
**Var. 5**	**Percentage of emergency team with EMT to all emergency teams (%)**	0.54	0.42	-0.31	-0.31	1.00																
**Var. 6**	**Kilometres of general road per square kilometre**	0.78	0.26	-0.42	-0.51	0.41	1.00															
**Var. 7**	**Kilometres of highway road per square kilometre**	0.38	0.29	-0.28	-0.25	0.42	0.46	1.00														
**Var. 8**	**Total kilometres of all roads per square kilometre**	a0.86	0.35	-0.43	-0.53	0.48	a0.97	0.50	1.00													
**Var. 9**	**Kilometres of impassable road per square kilometre**	0.57	-0.04	-0.19	-0.30	0.23	a0.80	0.20	0.79	1.00												
**Var. 10**	**Average coverage area per tertiary care centre**	-0.47	-0.38	0.57	0.53	-0.59	-0.54	-0.43	-0.56	-0.38	1.00											
**Var. 11**	**Average coverage area per fire station**	-0.60	-0.42	0.43	0.53	-0.31	-0.63	-0.46	-0.67	-0.49	0.58	1.00										
**Var. 12**	**Average coverage area per ambulance**	-0.64	-0.50	0.65	0.69	-0.32	-0.71	-0.49	-0.75	-0.50	0.68	a0.82	1.00									
**Var. 13**	**Average coverage are per emergency team**	-0.64	-0.49	0.63	0.68	-0.29	-0.72	-0.49	-0.75	-0.51	0.67	a0.86	a0.99	1.00								
**Var. 14**	**Average coverage area per emergency team with EMT**	-0.61	-0.48	0.65	0.66	-0.67	-0.68	-0.52	-0.72	-0.48	a0.82	0.75	a0.87	a0.85	1.00							
**Var. 15**	**Average coverage area per EMT**	-0.60	-0.57	0.76	0.77	-0.56	-0.64	-0.48	-0.69	-0.42	0.76	0.73	a0.92	a0.90	a0.94	1.00						
**Var. 16**	**Average coverage area per emergency crew**	-0.62	-0.47	0.59	0.64	-0.33	-0.70	-0.47	-0.73	-0.52	0.74	a0.86	a0.93	a0.95	a0.86	a0.88	1.00					
**Var. 17**	**Average coverage area per emergency medical specialist**	-0.46	-0.45	0.45	0.48	-0.52	-0.47	-0.33	-0.51	-0.31	0.69	0.48	0.58	0.55	0.66	0.67	0.57	1.00				
**Var. 18**	**Average coverage area per tertiary hospitals**	-0.49	-0.39	0.60	0.61	-0.52	-0.57	-0.43	-0.60	-0.38	0.67	0.65	0.70	0.69	0.77	0.77	0.73	0.55	1.00			
**Var. 19**	**Average coverage area per secondary hospitals**	-0.55	-0.40	0.58	0.58	-0.40	-0.61	-0.42	-0.64	-0.47	0.66	0.59	a0.82	a0.80	0.78	a0.85	0.78	0.59	0.64	1.00		
**Var. 20**	**Average cover area per primary hospitals**	-0.33	-0.31	0.45	0.43	-0.32	-0.42	-0.36	-0.43	-0.25	0.37	0.32	0.51	0.49	0.52	0.61	0.43	0.52	0.54	0.68	1.00	
**Var. 21**	Population density	a0.91	0.71	-0.42	-0.45	0.52	0.71	0.39	0.78	0.44	-0.44	-0.53	-0.59	-0.58	-0.56	-0.57	-0.58	-0.43	-0.48	-0.48	-0.30	1.00

^a^Coefficient correlation of >0.8.

**Table 7:** Spearman's correlation among variables

**Spearman's correlation**																						
Label	*Variables*	**Var. 1**	**Var. 2**	**Var. 3**	**Var. 4**	**Var. 5**	**Var. 6**	**Var. 7**	**Var. 8**	**Var. 9**	**Var. 10**	**Var. 11**	**Var. 12**	**Var. 13**	**Var. 14**	**Var. 15**	**Var. 16**	**Var. 17**	**Var. 18**	**Var. 19**	**Var. 20**	**Var.21**
**Var. 1**	**Population**	1.00																				
**Var. 2**	**Pavement ratio (%)**	0.03	1.00																			
**Var. 3**	**Size(km^2^)**	0.11	-0.57	1.00																		
**Var. 4**	**Mountain area(km^2^)**	-0.16	-0.46	a0.85	1.00																	
**Var. 5**	**Percentage of emergency team with EMT to all emergency teams (%)**	0.37	0.31	-0.29	-0.36	1.00																
**Var. 6**	**Kilometres of general road per square kilometre**	0.58	0.05	-0.45	-0.55	0.34	1.00															
**Var. 7**	**Kilometres of highway road per square kilometre**	0.36	0.07	-0.25	-0.27	0.37	0.54	1.00														
**Var. 8**	**Total kilometres of all roads per square kilometre**	0.65	0.11	-0.44	-0.55	0.40	a0.98	0.55	1.00													
**Var. 9**	**Kilometres of impassable road per square kilometre**	0.60	-0.11	-0.22	-0.28	0.25	0.71	0.32	0.75	1.00												
**Var. 10**	**Average coverage area per tertiary care centre**	-0.51	-0.35	0.59	0.68	-0.65	-0.68	-0.45	-0.70	-0.51	1.00											
**Var. 11**	**Average coverage area per fire station**	-0.64	-0.38	0.50	0.66	-0.47	-0.70	-0.47	-0.76	-0.59	0.74	1.00										
**Var. 12**	**Average coverage area per ambulance**	-0.60	-0.40	0.60	0.71	-0.43	a-0.80	-0.49	a-0.83	-0.59	0.77	a0.87	1.00									
**Var. 13**	**Average coverage are per emergency team**	-0.58	-0.40	0.61	0.71	-0.42	a-0.81	-0.51	a-0.84	-0.60	0.77	a0.88	a0.99	1.00								
**Var. 14**	**Average coverage area per emergency team with EMT**	-0.58	-0.41	0.59	0.72	-0.67	-0.76	-0.52	a-0.80	-0.56	a0.86	a0.87	a0.93	a0.93	1.00							
**Var. 15**	**Average coverage area per EMT**	-0.52	-0.54	0.67	0.78	-0.61	-0.71	-0.52	-0.76	-0.51	a0.83	a0.83	a0.93	a0.92	a0.96	1.00						
**Var. 16**	**Average coverage area per emergency crew**	-0.57	-0.35	0.61	0.72	-0.42	-0.77	-0.47	-0.78	-0.60	a0.82	a0.85	a0.92	a0.93	a0.90	a0.88	1.00					
**Var. 17**	**Average coverage area per emergency medical specialist**	-0.48	-0.47	0.56	0.66	-0.59	-0.57	-0.41	-0.62	-0.42	0.74	0.70	0.78	0.77	0.79	a0.85	0.75	1.00				
**Var. 18**	**Average coverage area per tertiary hospitals**	-0.40	-0.33	0.70	0.76	-0.55	-0.70	-0.46	-0.69	-0.46	a0.89	0.76	a0.81	a0.81	a0.85	a0.83	a0.85	0.75	1.00			
**Var. 19**	**Average coverage area per secondary hospitals**	-0.59	-0.33	0.55	0.66	-0.42	-0.76	-0.40	a-0.80	-0.65	0.66	0.74	a0.86	a0.85	0.80	a0.84	0.78	0.70	0.68	1.00		
**Var. 20**	**Average cover area per primary hospitals**	-0.29	-0.34	0.56	0.58	-0.42	-0.64	-0.52	-0.66	-0.30	0.45	0.49	0.64	0.62	0.59	0.66	0.48	0.60	0.54	0.67	1.00	
**Var. 21**	Population density	0.64	0.44	-0.61	-0.74	0.54	0.79	0.51	a0.85	0.56	a-0.83	a-0.88	a-0.96	a-0.95	a-0.95	a-0.96	a-0.90	a-0.80	a-0.82	a-0.88	-0.64	1.00

^a^Coefficient correlation of >0.8.

1) Population: "total kilometres of all roads per square kilometre" and "population density"

2) Size (km^2^): "mountain area (km^2^)"

3) Kilometres of general road per square kilometre: "total kilometres of all roads per square kilometre", "kilometres of impassable road per square kilometre", "average coverage area per emergency team", and "average coverage area per ambulance"

4) Total kilometres of all roads per square kilometre: "average coverage area per ambulance", "average coverage are per emergency team", "average coverage area per emergency team with EMT", "average coverage area per secondary hospitals" and "population density"

5) Average coverage area per tertiary care centre: "average coverage area per emergency team with EMT", "average coverage area per EMT", "average coverage area per emergency crew", "average coverage area per tertiary hospitals" and "population density"

6) Average coverage area per fire station: "average coverage area per ambulance", "average coverage area per emergency team", "average coverage area per emergency crew" and "population density"

7) Average coverage area per ambulance: "average coverage area per emergency team", "average coverage area per emergency team with EMT", "average coverage area per EMT", "average coverage area per emergency crew", "average coverage area per tertiary hospitals", "average coverage area per secondary hospitals" and "population density"

8) Average coverage area per emergency team: "average coverage area per emergency team with EMT", "average coverage area per EMT", "average coverage area per emergency crew", "average coverage area per tertiary hospitals", "average coverage area per secondary hospitals" and "population density"

9) Average coverage area per emergency team with EMT: "average coverage area per EMT", "average coverage area per emergency crew", "average coverage area per secondary hospitals", "average coverage area per tertiary hospitals" and "population density"

10) Average coverage area per EMT: "average coverage area per emergency crew", "average coverage area per emergency medical specialist", "average coverage area per tertiary hospitals", "average coverage area per secondary hospitals" and "population density"

11) Average coverage area per emergency crew: "average coverage area per tertiary hospitals" and "population density".

Therefore, variables with correlation coefficient of more than 0.8 which were excluded from the regression analysis were: "population density", "total kilometres of all roads per square kilometre", "mountain area", "kilometres of impassable road per square kilometre", "average coverage area per emergency team", "average coverage area per ambulance", "average coverage area per emergency team with EMT", "average coverage area per emergency crew", "average coverage area per EMT", and "average coverage area per tertiary hospitals" (Table 6 and 7).

### Multiple regression analysis

Stepwise (forward-backward) selection was performed in order to refine variables contributing to the model (Table 8). Variables were selected in the order where all variables in the model would reach a statistically significant level (*p *= 0.150), and would not be at a statistically significant level (*p *= 0.150) when other variables were added into the model. Consequently, "average coverage area per tertiary care centre", "kilometres of highway road per square kilometre", and "population" were selected. As a result of multiple regression analysis with these variables (Table 9), adjusted *R*^2 ^= 0.7001 (Table 10). The final results showed that the larger the average coverage area per tertiary care centre, the longer the travel time to the tertiary care centre. In addition, we found that the longer the highway road in kilometres per size of prefecture in square kilometres, the shorter the average travel time to the tertiary care centre; the larger the population, the shorter the average time to the tertiary care centre (Table 11).

**Table 8:** Stepwise selection

	*R*^2^	Adjusted *R*^2^	*C*(p)	*F *value	Pr>*F*
Average coverage area per tertiary care centre	0.5667	0.5667	22.3181	57.54	<0.0001
Kilometres of highway road per square kilometre	0.1054	0.6721	8.6749	13.82	0.0006
Population	0.0480	0.7201	3.5429	7.21	0.0103

**Table 9:** Analysis of variance model

Source	d.f.	Sum of squares	Mean square	*F *value	Pr>*F*
Model	3	12470	4156.58668	36.02	<0.0001
Error	42	4846.26077	115.38716		
Corrected total	45	17316			

**Table 10:** Index of goodness of fit

SD	10.74184
Mean of the dependent variable	56.83133
Coefficient of variance	18.90127
*R*^2^	0.7201
Adjusted *R*^2^	0.7001

**Table 11:** Results of multiple regression analysis

	d.f.	Parameter estimate	Standard error	*t *value	Pr>|*t*|
Intercept	1	61.19605	5.42175	11.29	<0.0001
Average coverage area per tertiary care centre	1	0.00441	0.00085607	5.15	<0.0001
Kilometres of highway road per square kilometre	1	-441.31025	133.82690	-3.30	0.0020
Population	1	-0.00000195	7.266741E-7	-2.69	0.0103

### Linear regression

**Figure 6:**
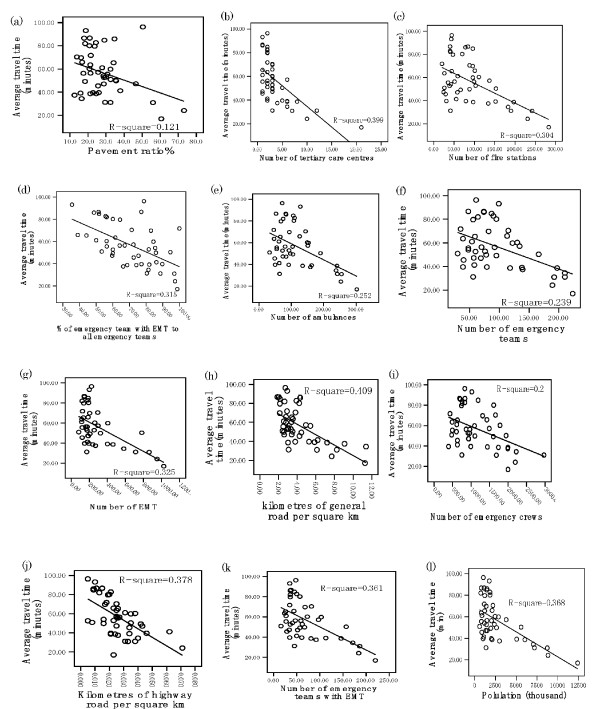
**Linear regression between average travel time and(a) pavement ratio**: *y *= -0.5483*x *+ 72.324 (*p *= 0.0178), *x *= 26; (b) number of tertiary care centres: *y *= -3.4802*x *+ 69.466 (*p *= 0.0000), *x *= 3; (c) number of fire stations: *y *= -0.1711*x *+ 72.042 (*p *= 0.0001), *x *= 83; (d) percentage of emergency team with EMT for all emergency teams: *y *= -0.6629*x *+ 103.579 (*p *= 0.0001), *x *= 69; (e) number of ambulances: *y *= -0.1484*x *+ 73.859 (*p *= 0.0004), *x *= 108; (f) number of emergency teams: *y *= -0.1825*x *+ 74.321 (*p *= 0.0006), *x *= 90; (g) number of EMT: *y *= -0.0483*x *+ 70.484 (*p *= 0.0000), *x *= 262; (h) kilometres of general road per square kilometre: *y *= -5.4808*x *+ 79.819 (*p *= 0.0000), *x *= 4.02; (i) number of emergency crew: *y *= -0.0148*x *+ 74.148 (*p *= 0.0019), *x *= 1102; (j) kilometres of highway road per square kilometre: *y *= -891.5370*x *+ 79.895 (*p *= 0.0000), *x *= 0.024; (k) number of emergency teams with EMT: *y *= -0.2283*x *+ 72.969 (*p *= 0.0000), *x *= 66; (l) population: *y *= -0.005*x *+ 69.155 (*p *= 0.0000), *x *= 2279.

As a result of the single regression analysis, linear regression analysis was performed between the average travel time and the variables. We constructed linear regression equations (*y *= *a *+ *bx*), where *x *is the emergency transfer system and *y *is the average travel time. The *x *value of each regression equation was calculated (Figure 6). The "average coverage area per tertiary care centre", one of the variables selected in the multiple regression analysis, is not shown in the linear regression analysis, as this variable showed a positive *t *value as a result of the single regression analysis (Table 5).

## Discussion

The introduction of GIS in our study made it possible to examine the average travel time to tertiary care centres, as well as the regional gaps throughout Japan. Our findings clarified the factors that would impact on decreasing average travel time to tertiary care centres: decrease in the average coverage area per tertiary care centre, extension of highway road per square kilometre, and increase in the population. Improvements on these systems should be emphasized rather than other aspects of the emergency transfer system. To ensure decrease of average coverage area per tertiary care centre, increasing the number of tertiary care centres is one strategy. Re-evaluation of the current locations of tertiary care centres, especially in prefectures with relatively few tertiary care centres compared to size, may be necessary. A survey by the MHLW found that prefectures with relatively large coverage area per tertiary care centre did not necessarily have low admission rates [15]. Thus, further discussion is necessary on whether the expansion of tertiary care centres is realistic in smaller districts. At the same time, re-evaluation of effective locations of tertiary care centres, as well as the concentration of specialists within centres is required. As a relatively new development, "new-type tertiary care centres", with smaller number of beds than traditional tertiary care centres, have been developed as a result of easing of establishment standards [16]. In conjunction with the findings of this study, more of these "new-type" tertiary care centres need to be established, especially in regions with large coverage area per tertiary care centre. The establishment of centres tailored to local circumstances is necessary. On the other hand, our findings also showed that larger populations lead to shorter average travel time. This implies that in larger cities, average travel times to tertiary care centres are shorter. However, from the aspects of policy-making, the increase in the population may be difficult to implement.

The other strategies that support our findings are the development of highway roads and national roads. Hashimoto et al. [5] established a model relating to the reduction of travel time as a result of road development, and estimated the number of people that could potentially be saved. Another strategy is the introduction of an emergency medical helicopter service. Wakayama prefecture, for example, was ranked 46th out of all 47 prefectures in travel time, because of its geographical characteristics, located along a peninsula in an elongated manner from north to south. As such, measures for introducing a helicopter service [17] and the construction of highways encompassing the surrounding prefectures [18] have recently been considered and implemented in Wakayama.

In addition, our findings have generated benchmarks for the emergency transfer system that could be effective in reducing the average travel time to tertiary care centres in prefectures with travel times longer than the average 57 min. Those benchmarks were: pavement ratio of roads, 26%; tertiary care centres, 3; fire stations, 83; emergency team with emergency medical technicians (EMT) in all emergency teams, 69%; ambulances, 108; emergency teams, 90; EMTs, 262; general road per km^2^, 4.02 km; emergency crews, 1102; highway road per km^2^, 0.024 km; emergency teams, 66; and population, 2279. Consideration of these benchmarks may be beneficial in the decision-making process of Medical Care Planning in each prefecture in order to reduce the average travel time to tertiary care centres in prefectures with travel times longer than 57 min.

The demand for emergency medicine in Japan has been increasing; in 1996 the number of emergency patients transferred to hospitals was 324,000, whereas in 2003 that number increased to 457,000 [6]. The number of patients with serious conditions was 6.5 per day per 0.8–1 million capita [19], and of 1000 patients per day, 450 were admitted to tertiary care centres [15]. In Nagasaki prefecture located in the south of Japan, 46% of emergency patients were over 65 years [5]. As the aging of the population continues, demand for emergency medicine will increase. Therefore, it is even more essential to reduce the travel time from the first response to the patient's admission to the hospital.

In Japan, focus on the need for emergency medicine coincided with the formulation of the Government's Medical Care Planning around 1985. At that time, establishment and development of the health care system was designed with focus on quantity. Thus, one of the drawbacks of such efforts is that resources regarding emergency medicine are not efficiently allocated. This may result in some situations where tertiary care is not accessible. As such, while the emergency medical system in Japan has been developed, regional gaps by prefectures were found in the number of emergency medical specialists as well as facilities [20]. The lack of medical resources has resulted in rejection of care by hospitals, or in other situations, the patient being bounced from one hospital to the next [21], which causes delay and, at times, dire outcomes for the patients. In particular, a patient with a time-critical injury who needs care from a specialist in a tertiary care centre would be adversely affected by having difficulty in access to such a centre [22].

This study analysed equitable access to emergency care; thus the issue of quality has not been examined. As adequate quality as well as quantity of health care provision is necessary for equitable access to emergency care, further studies on quality are required.

As the population of Japan is rapidly aging, the characteristics of diseases are changing correspondingly. Thus, the emergency medical system requires further research to ensure equitable care going forward. We believe the results of our study provide beneficial information to health policy administrators in formulating Medical Care Planning and, in general, in the decision-making process for health policy. By enhancing the emergency transfer system as we have set out here, travel time to emergency care would be reduced, ultimately leading to an improvement in the survival rate of emergency patients.

### Limitations of this study

Official data on the outcome of emergency medical patients is not available. Specifically, the survival rate and the number of patients transferred to tertiary care centres have not been disclosed. Thus we performed this research using officially available data, such as the development of roads and the status of the emergency transfer system, as factors that impact travel time. In the near future, the collection and disclosure of such outcome data will be desirable. One reason for the unavailability of outcome data could be because the emergency transfer system is dual-administered by the FDMA and the MHLW. If this dual system is generating inefficiencies, further review of this issue may be necessary.

**Figure 7:**
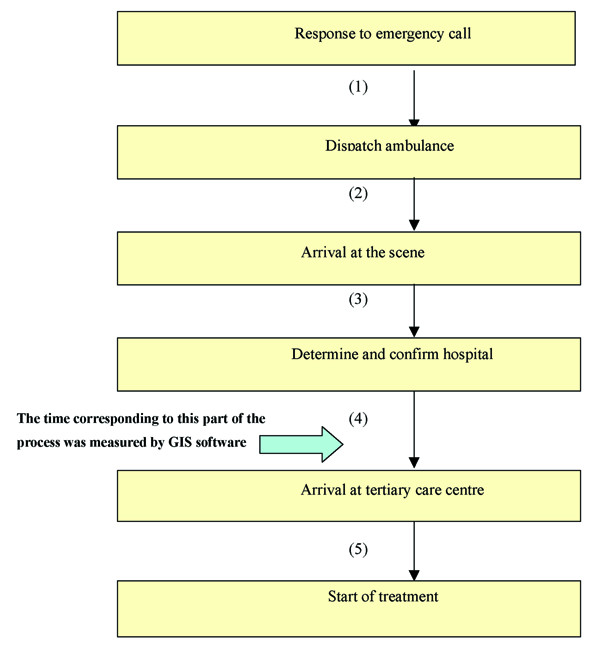
Process of the emergency transfer system.

In this research, "travel time" represents only Process 4 of the emergency transfer system (Figure 7(4)); this is a limitation that makes comparison between this study and other studies difficult. If actual travel time of all the processes in the emergency system were to be measured for each prefecture, and by each emergency level, it would significantly increase the accuracy of the data on average travel time to emergency hospitals, including tertiary care centres. This would make comparison between studies easier, even studies in other countries and would also offer an opportunity to re-examine the emergency transfer system throughout the country, e.g. further development of emergency transfer system focusing on quantity. Further research is necessary to collect actual travel times for the other processes, leading to a more accurate and comprehensive understanding of the emergency transfer system.

In this analysis, correlations between variables were extremely high, so we anticipated that there was a high risk of multicollinearity if multiple regression analysis was performed without selecting variables. Therefore, variables were selected based on the results of correlation analysis. However, in this process of selecting variables, there was also the risk of excluding variables that were the main factors on the average travel time from the analysis. In principle, when strong correlation is found between variables, causal association cannot be proved unless intervention studies are carried out. Nevertheless, by evaluating the time-series change in the data, inference of causal association is possible to a certain degree. Consequently, in future analysis, it would be desirable to refine the analytical results by considering the time-series changes for each variable.

## Conclusions

Our research has identified regional gaps in accessibility to tertiary care centres in Japan. Our findings have also clarified where in the emergency transfer systems we should place priority in terms of effectively reducing travel time. Further reduction of travel time to tertiary care centres can be effectively and efficiently achieved by improving upon these systems as set out in this paper. Benchmarks for the emergency transfer system have been generated that could be effective in reducing the average travel time to tertiary care centres in prefectures with travel times longer than the average 57 min.

This paper provides information to serve as a basis in providing equality of access to emergency medicine. Measures for reducing travel time need to be considered by policy-makers to re-evaluate the current locations of tertiary care centres, in order to provide universally accessible emergency medical care.

## Methods

### Statistical samples

All 47 prefectures in Japan were included in the sample for measuring average travel time. We performed statistical analysis using variables related to the development of the emergency transfer system and road conditions.

### Variables

The variables used in the analysis are shown in Table 12. These 23 variables were candidates as factors related to the transfer system and the condition of the roads, which impact on the average travel time to tertiary care centres. Pavement ratio is the percentage of kilometres of paved roads to kilometres of general roads. Roads are categorized into general roads and express roads. General roads include general national highways, prefectural highways and municipal roads.

**Table 12:** Distribution of each variable

Variables	Mean	Median	SD
Population^a^	2716744.68	1769000.00	2571553.95
Average travel time (minutes)	57.76	55.52	20.42
Pavement ratio (%)^b^	28.13	25.60	12.34
Size(km^2^)^c^	7739.15	5761.00	11701.22
Kilometres of general road^b^	23913.19	19448.00	14270.90
Kilometres of express road^b^	153.15	133.00	100.63
Mountain area(km^2^)^d^	4900.64	3820.00	5994.43
Total kilometres of all roads^b^	25161.57	23167.00	15144.02
Road area (km^2^)^b^	150.81	136.43	104.14
Kilometres of impassable road^b^	3495.96	2343.00	3741.87
Maintenance ratio of general road (%)^b^	53.94	53.30	0.12
Number of tertiary care centres^e^	3.72	3.00	3.58
Number of fire stations^f^	96.66	78.00	82.14
Percentage of emergency team^f ^with EMT to all emergency teams (%)	70.34	71.30	16.47
Number of ambulances^f^	119.91	94.00	74.75
Number of emergency teams^f^	100.23	79.00	60.13
Number of emergency teams with EMT^f^	73.17	50.00	53.85
Number of EMT^f^		189.00	249.91
Number of emergency crew^f^	1232.68	968.00	743.49
Number of emergency medical specialists^e^	41.11	21.00	54.00
Number of tertiary hospitals^g^	4.32	3.00	3.64
Number of secondary hospitals^g^	76.49	52.00	65.85
Number of primary hospitals^g^	36.91	28.00	30.88

*N *= 47 for all variables. EMT, emergency medical technician.

^a^Ministry of Internal Affairs and Communications, Statistics Bureau 2004.

^b^National Land and Transportation Ministry 2003.

^c^Geographic Institute Survey 2005.

^d^National Land and Transportation Ministry 1983.

^e^Japanese Association for Acute Medicine, as of February 2005.

^f^Fire and Disaster Management Agency, Ministry of Internal Affairs and Communications 2004.

^g^Survey of Health Care Facility, 2002 Ministry of Health, Labour and Welfare.

### Process of analysis

In determining the variables that impact on average travel time (minutes), and to ultimately perform multiple regression analysis, the selection of variables was conducted as follows:

(1) To determine if the variables should be adjusted for size (km^2^) or population, or be kept unadjusted, correlation analysis (Spearman and Pearson) between population and size (km^2^) with each variable was performed. Based on the correlation analysis, the variables used for regression analysis of average travel time were selected. Hokkaido was excluded from the analysis as an outlier.

(2) Single regression analysis of the average travel time was performed using variables selected from the correlation analysis.

(3) Correlation analysis between variables was performed using the variables selected for the single regression analysis, and candidate variables for multiple regression analysis were refined.

(4) Stepwise selection was performed in order to refine variables contributing to the model. Consequently, multiple regression analysis was performed using selected variables and factors impacting average travel time.

(5) As a result of the single regression analysis in (2), linear regression was performed to model the relationship between the average travel time and variables with negative *t *values, and statistical significance. This was performed in order to estimate the benchmarks in the emergency transfer system that would be effective in reducing the average travel time to tertiary care centres in prefectures with travel times longer than the average 57 min.

SAS9.1.3 and the SPSS 12.0 were used for the statistical analyses.

### Calculation of travel time

This paper examined and modelled average travel time (minutes) to tertiary care centres in Japan. Using GIS software (Market Planner GIS version 2.1, Pasco Corporation ArcView 9), we constructed geographical plots representing the centroids of 2594 municipalities (designated by the Geographic Survey Institute, April 2005) and tertiary care centres. Based on travel distance (not straight line distance) using road network information and average travel speed (as observed by the National Land and Transportation Ministry), travel times (minutes) to tertiary care centres were calculated. Consequently, travel distance (km) and travel time (minutes) from the centroid of all municipalities to the nearest tertiary care centre were estimated. Travel times to tertiary care centres in each municipality were then aggregated by the 47 prefectures, and the average travel time (minutes) for each prefecture was calculated.

### Types of travel time

Travel time (minutes) used in this research does not include the whole time required in the full procedure of the emergency transfer system, which is measured as the time required from the first response to the emergency call to the start of treatment after the patient is transferred to the tertiary care centre. The emergency transfer system in Japan is coordinated by both the fire department system and the emergency medical system. Specifically, once computer-assisted emergency dispatch operators respond to emergency calls, the nearest available ambulance is dispatched from the fire station to the scene of the incident. When the ambulance arrives at the emergency scene, the emergency crew determine the nearest available hospital and the ambulance will go to that hospital. In this study, travel time is defined as the time taken from the determination and confirmation of hospital to arrival at the tertiary care centre. As shown in Figure 7, what we did not include is the time required from the response to the emergency call to the dispatch of the ambulance (Figure 7(1)), from the dispatch of the ambulance to arrival at the scene (Figure 7(2)), from arrival at the scene to the determination and confirmation of hospital (Figure 7(3)), and from arrival at tertiary care centres to the start of treatment (Figure 7(5)).

### Transportation means

We estimated the travel time assuming the use of private car or ambulance. Public transport, such as railway or bus, and other means of transport such as helicopters were not included in this study. Generally speaking, in most cases of emergency transfer, the percentage transported by ambulance was 99.9%, and helicopter transport accounted for 0.04% [6]. In remote locations where an ambulance is unavailable, patients are mostly transported by private car. Therefore, we believe the scenario we have assumed in this study, i.e. transportation by private car or ambulance, is sufficient.

Using the above-mentioned travel time, our study was undertaken to identify:

(1) the average travel time (minutes) to a tertiary care centre for all prefectures in Japan and the regional gaps;

(2) the effects of development of the emergency transfer system and the effect that improvements in road conditions would have on the travel time (minutes) to tertiary care centres;

(3) benchmarks for the emergency transfer system that would be effective in reducing the average travel time (minutes) to tertiary care centres in prefectures with travel times longer than the average 57 min.

## Competing interests

The author(s) declare that they have no competing interests.

## Authors' contributions

MM collected data and carried out the analysis. KH organized the collection of information on tertiary care centres. AH collaborated in the development of the paper. KK organized the management of the study.
